# Enhanced rhamnolipid production by *Pseudomonas aeruginosa* overexpressing *estA* in a simple medium

**DOI:** 10.1371/journal.pone.0183857

**Published:** 2017-08-24

**Authors:** Leticia Dobler, Bruna Rocha de Carvalho, Wilber de Sousa Alves, Bianca Cruz Neves, Denise Maria Guimarães Freire, Rodrigo Volcan Almeida

**Affiliations:** 1 LaMMP–Laboratório de Microbiologia Molecular e de Proteínas, Chemistry Institute, Federal University from Rio de Janeiro, Rio de Janeiro, Brazil; 2 LaBiM–Laboratório de Biotecnologia Microbiana, Chemistry Institute, Federal University from Rio de Janeiro, Rio de Janeiro, Brazil; Washington State University, UNITED STATES

## Abstract

A modified *Pseudomonas aeruginosa* strain capable of overexpressing the estA gene, an encoding gene for a membrane-bound esterase, was constructed and its rhamnolipid (RML) production was studied. Fermentations using wild-type (WT) and modified *P*. *aeruginosa* strains were conducted until exhaustion of glycerol in Medium Salt Production, using two different C/N ratios. At a C/N of 83.2, the modified strain produced up to 3.9 times more RMLs than the WT, yielding a maximum concentration of 14.62 g/L RML when measured by HPLC and 22 g/L by the orcinol assay. Cell-free supernatant from the modified strain reduced surface tension to 29.4 mN/m and had a CMC of 240 mg/L and CMD of 56.05. This is the first report on the construction of an estA-based recombinant strain for RML production.

## Introduction

Surfactants are surface-active molecules that are able to lower the surface and interfacial tension between phases. Although biosurfactants have several advantages over chemical surfactants, their production is still costly and the production process still requires further optimization. Among the biosurfactants of microbial origin, rhamnolipids (RML) are a very important group of molecules [1–3] with potential application in a variety of industrial sectors, such as the pharmaceutical, cosmetic, food and oil industry [2] with a growing interest during last years as in the scientific literature as in patents [3].

*P*. *aeruginosa* is the most widely used microorganism for RML production [1], despite being an opportunistic pathogen. So far, there is a shortage of non-pathogenic strains capable of achieving the same RML production levels as strains from the *Pseudomonas* genus [3,4]. The models and tools for fermentation processes in bioreactors are better described for *P*. *aeruginosa* PAO1, making this strain a common choice for studies on a bench scale [5].

The discovery of EstA, an esterase anchored on the outer membrane of *P*. *aeruginosa* PAO1 [6], has attracted attention in a broad field of study. Since its discovery, EstA has attracted growing interest as a cell surface anchor [7–9], which has motivated the determination of its structure [10]. EstA is classified as an auto-transporter protein, within a family of type V secretion systems, and has been studied in order to elucidate how these proteins can translocate to the outer membrane [11,12].

In 2007, EstA was related to cell motility, biofilm formation, and production of RMLs [13]. Thus far, no further research work has been reported involving this membrane-bound enzyme and RML production.

There are many studies in the literature on the construction of high-yield RML-producing strains. Mostly, they focus on modifying the genes that play a direct role in the RML biosynthesis pathway, such as *rhlA*, *rhlB* and *rhlC*. Very few researchers have invested in unrelated genes, which have also produced good yields of rhamnolipids. Besides the proven relationship between the *estA* gene and RML production, no strain-optimization work has ever focused on *estA* gene expression modification [3].

The present study explores the previously described relationship between EstA and RML production by constructing a *P*. *aeruginosa* strain that overexpress the gene estA, called *P*. *aeruginosa*-estA.

The comparison of RML production by wild-type *P*. *aeruginosa* PAO1 and mutant strains was carried out using different C/N ratios in a simple and low cost medium. Additionally, the RML production by *P*. *aeruginosa*-estA was studied under different C sources.

## Materials and methods

### Bacterial strains and culture conditions

The pUCP26-estA plasmid was constructed with the insertion of a full-length *estA* gene from *P*. *aeruginosa* PAO1 (GenBank® accession number AF005091) at the *Eco*RI and *Bam*HI restriction sites of the shuttle vector pUCP26 [14], kindly provided by Professor Herbert Schweizer from Colorado State University. Thus, a synthetic DNA fragment including the signal peptide and its own ribosomal binding site (Shine-Dalgarno) was inserted under the control of the *lac* promoter. The plasmid was designed *in silico* and later synthesized by Epoch BioLabs®. and transformed into *E*.*coli* JM109 competent cells (Promega) according to the manufacturer's instructions, generating the strain *E*.*coli* JM109-estA. For the construction of the *P*. *aeruginosa-estA* strain, the shuttle vector pUCP26estA was transformed into *P*. *aeruginosa* PAO1 by heat shock, as previously described [15]. All strains were stored at -80°C in sterile solutions of 30 to 50% glycerol (Table 1). With the exception of the RML-producing medium, all liquid cultures were grown in Luria-Bertani (LB) broth (Sigma Aldrich). For the solid medium, 2% bacteriological agar (Vetec®) was added. Bacterial growth for the pre-inoculum was carried out in a liquid medium, in 50 mL conical bottom tubes, with 5 to 10 mL medium, rotation at 170 rpm, and 30°C.

**Table 1:** Strains and plasmids used at this study.

	Characteristics	Reference or Supplier
**Strains**		
*P*. *aeruginosa* PAO1	Wild-Type Amp ^R^	[16]
*P*. *aeruginosa-*estA	*P*. *aeruginosa* PAO1 containing plasmid pUCP26estA	This study
*E*.*coli* JM109	endA1, recA1, gyrA96, thi, hsdR17 (rk–, mk+), relA1, supE44, Δ (lac-proAB)	Promega®
*E*.*coli* JM109estA	*E*.*coli* JM109 with pUCP26estA	This study
**Plasmids**		
pUCP26	Tc ^R^, containing Ori from *Pseudomonas*, Ori from *E*. *coli*	[14]
pUCP26estA	pUCP26 containing the full-length *estA* from *P*. *aeruginosa* PAO1, including its native ribosomal-binding site	This study

### *P*. *aeruginosa*-estA fermentation with different carbon sources

Analyses of RML production by *P*. *aeruginosa-*estA were performed using seven different variations of MSP medium, consisting of: potassium phosphate buffer, magnesium sources, and a combination of carbon and nitrogen sources, as shown in Table 2.

**Table 2:** Composition of the production media (g/L).

C source		N source		K_2_HPO_4_	KH_2_PO_4_	MgSO_4_.7H_2_O
Glycerol	42.00	Sodium nitrate	1.40	7.00	3.00	0.20
Glycerol	42.00	Ammonium sulfate	1.09	7.00	3.00	0.20
Olive oil	21.53	Sodium nitrate	1.40	7.00	3.00	0.20
Soy oil	21.50	Sodium nitrate	1.40	7.00	3.00	0.20
Oleic acid	21.45	Sodium nitrate	1.40	7.00	3.00	0.20
Ethanol	31.50	Sodium nitrate	1.40	7.00	3.00	0.20
Glucose	41.05	Sodium nitrate	1.40	7.00	3.00	0.20

The masses of the carbon and nitrogen sources were calculated to achieve a C/N ratio of 83. All fermentations were carried out in duplicate at a C/N ratio of 83.2 (mol/mol). Final culture volumes of 100 mL were placed in 250 mL Erlenmeyer flasks for incubation. All fermentations were conducted at 30°C, with orbital agitation at 170 rpm. For all the experiments carried out using *P*. *aeruginosa-estA*, tetracycline was added to a final concentration of 100 μg/mL.

### Comparative analysis of *P*. *aeruginosa* PAO1 and *P*. *aeruginosa*-estA

For RML production, the transformed *P*. *aeruginosa*-estA and the wild strain *P*. *aeruginosa* PAO1 were inoculated in 1L flasks containing 300 mL of a growth medium consisting of 30.0 g/L glycerol, 1.4 g/L NaNO_3_, 7.0 g/L K_2_HPO_4_, 3.0 g/L KH_2_PO_4_, 0.2 g/L MgSO_4_.7H_2_O, 5 g/L soy peptone, and 5 g/L yeast extract. The cells were harvested at the exponential phase and inoculated at an initial cell concentration of 0.85 g/L (dry cell) in a 1 L flask containing 300 mL of two different fermentation media with different C/N ratios: MSP medium (7.0 g/L K_2_HPO_4_, 3.0 g/L KH_2_PO_4_, 0.2 g /L MgSO_4_.7H_2_O) at concentrations of 4.75 g/L of NaNO_3_ and 30 g/L of glycerol, resulting in a medium with a C/N ratio of 17.5 (mol/mol); and 1.4 g/L of NaNO_3_ and 42 g/L glycerol, resulting in a C/N of 83.2 (mol/mol). Fermentations were conducted until exhaustion of glycerol, and samples were withdrawn for biomass and glycerol concentration analyses, surface tension measurements, and the calculation of the critical micelle concentration (CMC).

The choice of 17.5 and 83.2 as the C/N ratios was based on the reports of Tavares et al. [17] and Santos et al. [18].

All the fermentations were conducted at 30°C with 170 rpm orbital agitation and at a dark room. For all the experiments carried out using *P*. *aeruginosa-estA*, tetracycline was added to a final concentration of 100 μg/mL.

### Bacterial cell quantification

A standard curve of cell growth was plotted using absorbance at 600 nm against dry mass concentration (g/L).

### Esterase activity assay

The wild types and constructed strains were tested for their esterase activity using 4-methylumbelliferyl-heptanoate (MUH) as a substrate. Activity in MUH was measured using a fluorometer, as described by Prim et al. [19], at 54.8°C. Three milliliter aliquots of 50 mM sodium phosphate buffer pH = 8, were added to 12 μL of a MUH solution (25 mM solubilized in ethylene glycol monomethyl ether). To start the reactions, 10 to 50 μL cell suspension was added. The *P*. *aeruginosa* cell suspension was prepared as described in section 2.1. (pre-inoculum).

The production of 4-methylumbelliferone was measured by fluorescence (λex = 323 nm; λem = 448 nm) under initial reaction conditions. Standard curves were plotted using solutions that varied from 82.8 to 1225.00 μM MUF (4-methylumbelliferyl) solubilized in 50 mM sodium phosphate buffer pH = 8. All experiments were analyzed in triplicate (n = 3). One unit of activity was defined as the amount of enzyme required to produce 1 μmol MUF per minute under the test conditions, and was expressed in U per g of dry mass. The natural degradation of the substrate by the action of temperature over time was monitored and discounted for activity calculations.

### Determination of RML and glycerol concentration

The quantification of RML was assessed by the indirect quantification of rhamnose. Rhamnose was quantified by the addition of 100 μL of a 10 M sulfuric acid solution to 1 ml supernatant samples, and the hydrolysis generating rhamnose and fatty acid was conducted at 100°C for 4 hours. The mixture was neutralized with 10 M NaOH, filtered with a 0.22 μm filter, and analyzed using high performance liquid chromatography (HPLC). An Aminex HPX-87H column (BioRad®) was used for the HPLC assays, using 5 mM acid sulfuric as the mobile phase at a rate of 0.6 mL/min and oven temperatures of 42°C to assess rhamnose and 68°C to assess glycerol. Some samples were also quantified by the orcinol assay [20,21], for the purposes of comparison with data in the literature. The procedure was followed as described by Koch et al. [21], with some modifications: 200 μL samples of culture supernatant were mixed with 1.8 mL orcinol reagent containing 53% acid sulfuric and 0.19 orcinol. The mixture was incubated at 80°C for 30 minutes and absorbance at 421 nm (rhamnose) was measured in a spectrophotometer (Pharmacia). For the conversion of rhamnose to rhamnolipid, a factor of 2.5 was used, on the assumption that 1 g rhamnose corresponds to 2.5 g rhamnolipid mixture [13,22,23].

### Thin-layer chromatography (TLC)

The RML mixture produced in each fermentation was analyzed by thin-layer chromatography (TLC), as described by Wilhelm et al. [13], with some modifications. Two microliters of each fermentation supernatant was applied 1cm up from the bottom of the silica sheet. Silica 60 gel aluminum sheets were used (Merck®, Germany). A mobile phase consisting of chloroform:methanol:glacial acetic acid (65:15:02) was added until 0.8 cm of the standing layer was reached. After the layer was dried, it was stained for 10 minutes in a chamber at 100°C using a buffer of a stained reagent, containing 75 mg orcinol, 4.2 mL concentrated sulfuric acid, and 21 mL deionized water, as reported by Tielen et al. [23]. The assay was done in duplicate.

### n-hexadecane emulsion

Two milliliters of final fermentation supernatant (free of glycerol) was mixed vigorously with 2 mL n-hexadecane (reference for fuel mixtures/cetane number 100) for 2 minutes. The mixture was left to stand and its emulsion formation capability was observed after 24 hours.

### Surface tension assay, CMC and CMD assessment

Surface tension was determined using the DSA 100S Drop Shape Analyzer (Krüss), as described by Song and Springer (1996). To assess CMC (critical micelle concentration) and CMD (critical micelle dilution), several dilutions were made of the cell-free fermented medium, from 0 to 1000. All the analyses were performed with the last-day fermentation sample, free of glycerol, as this compound could interfere with drop formation in the surface tension assay.

## Results and discussion

### Esterase expression in *P*. *aeruginosa*

**Fig 1:**
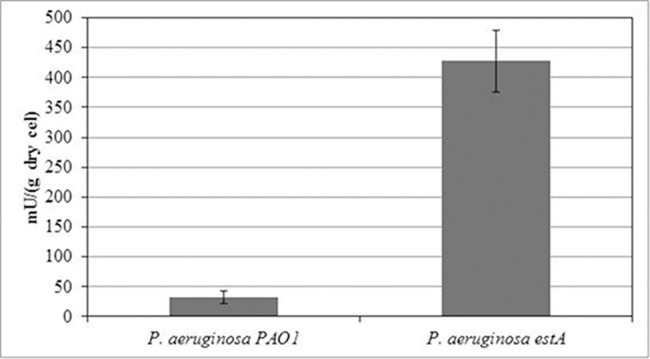
Esterase activity of wild type and recombinant strain. Recombinant strain was created by the insertion of pUCP26-estA plasmid, that contains *estA* gene, for encoding an outer membrane esterase. Esterase activities for the wild type *P*. *aeruginosa* PAO1 and the recombinant *P*. *aeruginosa*-estA were carried out using MUH (C7) as a substrate, at 30°C and pH = 7. A Student's t-test was performed and it is affirmed that the data are significantly different from each other (p<0.5).

Recombinant EstA expression in *P*. *aeruginosa* was confirmed by the 13-fold increase of esterase activity per gram of cell when the wild type (32.6 mU) was compared with the modified strain (427.8 mU) (Fig 1).

### RML production by *P*. *aeruginosa*-estA in different carbon sources

**Fig 2:**
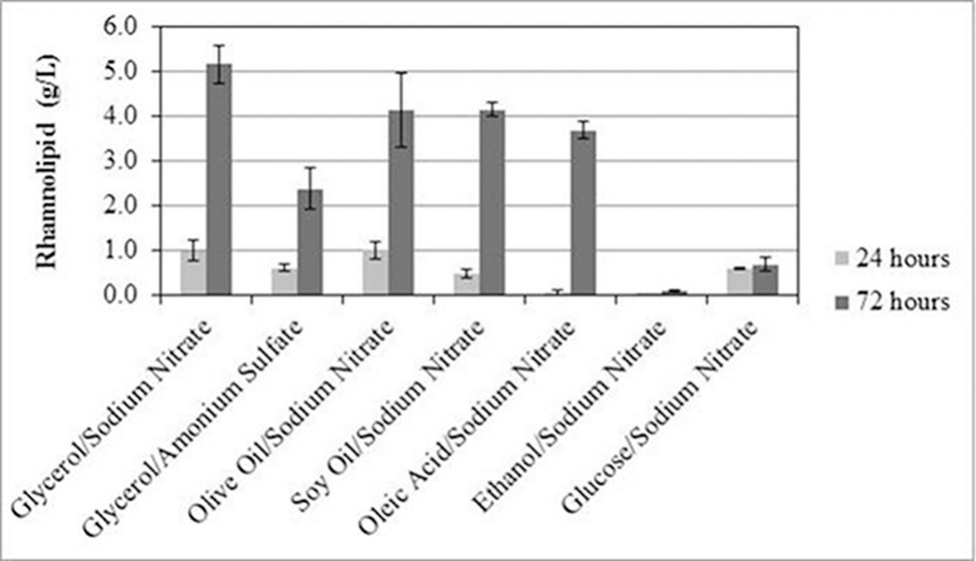
RML production using different carbon and nitrogen sources. Analyses of RML production by *P*. *aeruginosa*-estA were performed using several variations of MSP medium (glycerol, olive oil, soy oil, oleic acid, ethanol and glucose as carbon source and sodium nitrate and ammonium sulfate as nitrogen sources). Figure presents RML production after 24 (light gray) and 72 hours (dark gray). All cultives were conducted at C/N ratio of 83.2 (mol/mol).

In order to evaluate the production of the recombinant strain in different carbon sources, a comparative analysis of the RML production of *P*. *aeruginosa-estA* was performed in seven different MSP media with different carbon or nitrogen sources (Fig 2). The fermentation using sodium nitrate as a nitrogen source and glycerol as a carbon source had the highest yield, reaching 5.16 g/L RML in the first 72 hours. Ammonium N is assimilated faster than N from nitrate, as nitrate suffers dissimilatory nitrate reduction to ammonium. Santos et al., [18] discussed the participation of nitrogen in the production of RML by *Pseudomonas aeruginosa* PA1. As RML production is accentuated under nitrogen-limited conditions, the use of nitrate could be seen as a way of simulating such conditions, where the production of this biosurfactant is known to be higher.

Benicasa et al. [24] produced RML, under batch cultivation, using *Pseudomonas aeruginosa* LBI and soapstock as the sole carbon source. The soapstock was composed mainly of linoleic acid (50%) and oleic acid (25%), and reached a maximum rhamnolipid concentration of 15.9 g/L at 54h (productivity of 29.4mgRML/L.h) using the source at an initial concentration of 2.5%.

For any future research designed to investigate the *in-vivo* function of EstA, it will be important to study how a negative mutant for *estA* would behave in glycerol, triglyceride, and fatty acids. Also, it is broadly known that different oils used in fermentation will result in different congeners of RML [25], yielding different surface physicochemical properties.

Matsufuji et al. [26] produced up to 32 gRML/L using *Pseudomonas aeruginosa* IFO 3924 (a non-modified strain) in 7 days of cultivation fed with ethanol 55.3 g/L, achieving one of the highest final RML concentrations reported in the literature. However, this result was obtained only when fed-batch strategy was used. In a simple batch (with 30g/L ethanol in a complex media) the authors obtained 3.7 gRML/L. Santos et al. (18) using *Pseudomonas aeruginosa* PA1 obtained 2 gRML/L in a medium containing ethanol (30 g/L) and yeast extract (5 g/L), as carbon and nitrogen source respectively. Unfortunately, *P*. *aeruginosa-*estA was not able to produce RMLs using ethanol as a carbon source in the simple medium used in this research.

Likewise, glucose, one of most commonly used substrates for RML production [4], did not yield high amounts of RML. Wittgens et al. [4] produced up to 1.5 g/L RML, measured by the orcinol method. They used 5–10 g/L of glucose in the culture medium, while in our study the medium consisted of 41.05 g/L in order to keep the same C/N ratio for all the carbon sources. The low RML production and higher substrate concentration could be explained by the inhibition of the cell by the higher concentration of glucose.

Thus, we chose the low-cost byproduct from biodiesel refining, glycerol, as the best carbon source (together with sodium nitrate as a nitrogen source) for RML production, using the new engineered strain, *P*. *aeruginosa-estA*.

### Production of RML by *P*. *aeruginosa-estA* and *P*. *aeruginosa* PAO1 in MSP-glycerol medium

The comparative analysis of the modified and wild-type strains was performed in two different MSP media, using 30 and 42 g/L glycerol as the sole carbon source, at a C/N ratio of 17.5 and 83.2 (mol/mol), respectively. These fermentations were carried out in 300 mL MSP culture medium, and RML yields were assessed by HPLC.

**Fig 3:**
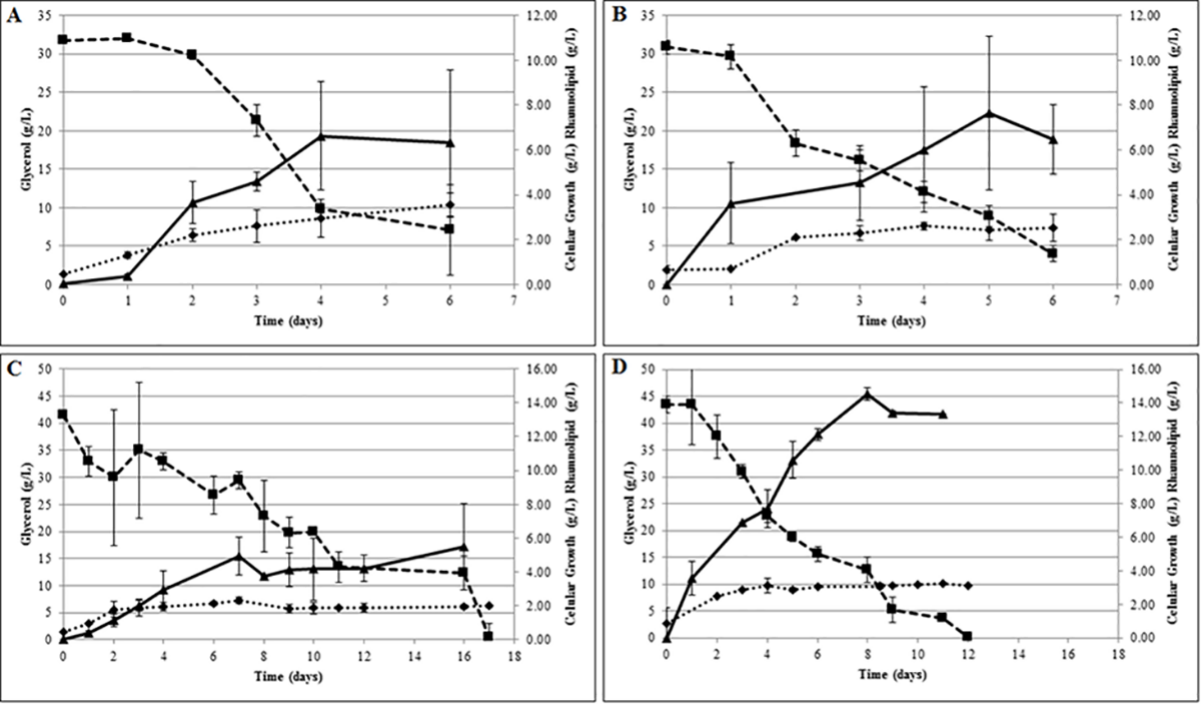
Kinetic of rhamnolipid production. Wild type and recombinant strains were used at cultives under MSP media in two different C/N ratios. Glycerol was used at carbon source and sodium nitrate as nitrogen source. RML production and glycerol consumption was accessed by liquid chromatography. General data on cell (rhombus), rhamnolipid (triangle), and glycerol (square) concentrations (g/L) are shown over time for A) wild-type strain (*P*. *aeruginosa* PAO1) at C/N = 17.5; B) modified strain (*P*. *aeruginosa*-estA) at C/N = 17.5; C) wild-type strain (*P*. *aeruginosa* PAO1) at C/N = 83.2; D) modified strain (*P*. *aeruginosa*-estA) at C/N = 83.2.

No difference was found between the strains in terms of their production when C/N = 17.5 (Fig 3A and 3B).

However, a difference of up to 290% was observed in RML when C/N = 83.2 (Fig 3C and 3D). Under these conditions, the *P*. *aeruginosa-estA* transforming strain was able to produce 14.6 g/L RML and 76.0 mg/L.h of productivity on the 8^th^ fermentation day, while the wild type produced 3.7 g/L RML and 19.3 mg/L.h. The maximum RML concentration for the wild strain was attained only on the 16^th^ day, when 5.5 g/L of RML and a productivity of 14.3 mg/L.h was obtained. The pH at the final day were 7±0.2 to all conditions. The modified strain exhausted the glycerol by the 12^th^ day, while the wild strain exhausted the glycerol by the 17^th^ day. The cell viabilities were maintained during these periods. The depletion of nitrogen probably occurred at the firsts days of culture, inhibiting the cell growth. In the other hand, as soon the glycerol keeps available, the existing cell can keep their availability and produce rhamnolipids

Table 3 compares all four fermentation conditions and the main fermentation parameters. The modified strain did not just produce more RML by time (productivity), but had a higher RML yield per biomass (Y_RLM/X_) for a C/N ratio of 83.2. Also, the mutant strain was able to better use its carbon source to produce RML (Y_RLM/S_) under the same conditions. The opposite was observed at C/N = 17.5, where the production of the wild-type and constructed strain had similar production by substrate consumption.

**Table 3:** Comparative biotechnological bioprocess parameters for four fermentations, using *P*. *aeruginosa* PAO1 and *P*. *aeruginosa-estA* and two different C/N ratios for the MSP medium (83.2 and 17.5 mol/mol). All data calculated for the day when production reached its maximum RML concentration.

Strain	C/N ratio	Time (day)	Max. Concentration (gRML/L)	Productivity (mgRML/h.L)	Y_RML/x (g/g)_	Y_RML/s (g/g)_	Y_x/s (g/g)_
**PAO1**	**83.2**	16	5.5	14.3	3.89	0.188	0.048
**PAO1**	**17.5**	4	6.6	68.8	2.66	0.300	0.113
***estA***	**83.2**	8	14.6	76.0	6,55	0.377	0.058
***estA***	**17.5**	6	6.5	45.0	3,45	0.235	0.068

These results show that the RML production by *P*. *aeruginosa*-estA strain is strongly influenced by the C/N ratio and do not often generate higher RML titer, as can be directly inferred in Whilhelm et al. (2007) (13).

The low productivity of the wild strain at C/N = 17.5 could be because the energy of the carbon source was channeled into cell growth (Y_x/s_), which reached 0.113 (g/g), while under the other condition it reached 0.068.As orcinol overestimates the RML concentration in a sample by recording other hexoses, it is difficult to compare the results with the data in the literature [3]. As such, samples of both the last day of fermentation and the day with maximum production were measured by orcinol assay to allow a better comparison. It was observed that the productivity of our modified strain was really higher than that of the other modified strains reported in the literature, as determined by both procedures (orcinol assay and HPLC analysis). In Table 4, we compare the results of the present findings with the main modified strains already reported in the literature [3], by both orcinol assay and HPLC analysis.

**Table 4:** Comparison between the transformed strains with the best production or productivity already reported in the literature and the results attained in this study, including one of best results achieved with a wild strain reported in the literature, for reference purposes.

Microorganism	Peculiarity	Maximum RLM (g/L)	Productivity (mg/L.h)	Carbon source(s)	Vf/Vm	Quantification Method	Reference
*P*. *aeruginosa* PAO1	Reference–wild type strain with great production	39	433	Sunflower oil (250g/L)	42L/19L	HPLC-UV/vis	[5]
***P*. *aeruginosa-estA***	***estA overexpression***	**14.6**	**76.0**	**Glycerol (42g/L)**	**1L/300mL**	**HPLC**	**This work**
***P*. *aeruginosa-estA***	***estA overexpression***	**22.0**	**114.6**	**Glycerol (42g/L)**	**1L/300mL**	**Orcinol**	**This work**
*P*. *aeruginosa* JC	*vhb* expression.	8.373	349	Glucose (10g/L) + Yeast extract (5g/L)	150/50mL	Phenolsulphuric	[27]
*P*. *putida* KCTC 1067 (pNE2)	*rhlA* and *rhlB* co-expression as an operon, clustered with *rhlR* and *rhlI*.	7.3	101.39	Soybean oil (20g/L)	NI	Orcinol	[33]
*P*. *aeruginosa* JC	*vhb* expression.	13.3	185	Raw cheese whey (500g/L)	250mL/50mL	Phenolsulphuric	[28]

NI—Not Informed

Our mutant strain reached a maximum of 14.6 g/L RLM when rhamnose was assessed by HPLC and 22g/L when the traditional orcinol assay was carried out. These concentrations are higher than any RML mutant reported in the literature [3].

Erenler and Kahraman [27] constructed the engineered strain *P*. *aeruginosa* JC, which is capable of heterologously expressing the *vhb* gene, which codes hemoglobin of the bacterium *Vitreoscilla*. The idea was to circumvent the difficulties generated by the oxygenation of the medium, which generates too much foam. By the expression of hemoglobin, the oxygen could be diluted at a higher rate in the medium and be more easily accessible for cell metabolism. The group achieved production of 8.373 g/L and productivity of 349 mg/L.h (measured by the phenol-sulfuric assay). The bioprocess presented by the group achieved high productivity, close to the most widely accepted model in the literature [5]. However, the low final RLM concentration is not suitable for good bioprocess design, because it generates costs in downstream processes. Also, production was carried out in a low volume (50 mL) and using a fairly expensive medium.

The same group later used the same strain with a raw, low-cost medium based on raw cheese whey, achieving a good concentration of RML (13.3 g/L), but the work still has some shortfalls. The concentration of waste in the culture medium was high (500 g/L), and productivity was lower than in the earlier study (185 mg/L.h). Also, the volume of production was still not scaled up (50 mL) [28].

To our knowledge, no engineered strain has achieved as high a final RML concentration as the ones reported for wild strains. Giani et al. [29] claimed a patent with maximum production of 112 g/L, which is the highest production reported in the literature. As no further work has been published since, the model described by Müller et al. [5,30] is recognized as the best for RML production.

Müller et al. [5] declare maximum production of 39 g/L and productivity of 433 mg/L.h using *P*. *aeruginosa* PAO1. However, these results were obtained in a bioreactor with control of temperature, pH, pO2 and foam. Also, it was applied a high amount of feedstock, 250 g/L of sunflower oil, a component more expensive than glycerol. Furthermore, despite the high final titer obtained by Müller et al. [5] the yield (Y_P/X_) was 2.02 gRML/g dry cell, while we present a yield of 6.55 gRML/g dry cell (Table 3). As the strain constructed in this research produced 290% more RML than *P*. *aeruginosa* PAO1, this leads us to believe that the present strain has significant potential for industrial applications.

### Characterization of RML produced

For the partial characterization of the RML specimens produced by the wild and mutated strains in different C/N ratio media, the RML mixture obtained on the last day of fermentation (free of glycerol) was studied by TLC, and its capability to emulsify n-hexadecane in water and its surface tension were measured.

**Fig 4:**
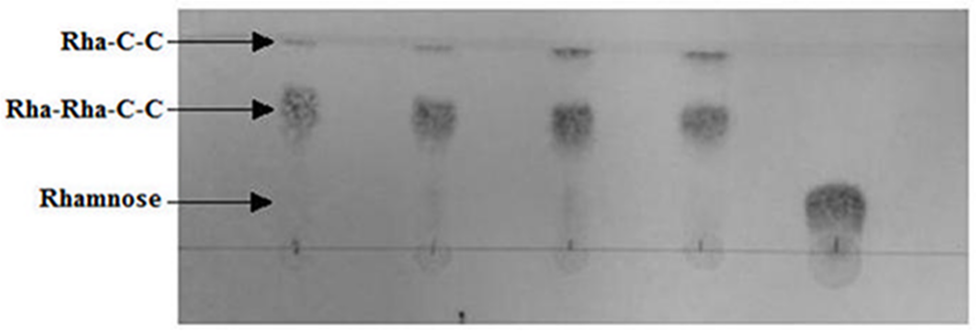
Thin-layer chromatography. In order to analyze the mono-dirhamnolipid ratio, a TLC was carried out by the application of 2μL of the final medium supernatant. From left to right: *P*. *aeruginosa* PAO1, C/N ratio = 17.5; *P*. *aeruginosa* PAO1, C/N ratio = 83.2; *P*. *aeruginosa*-estA, C/N ratio = 17.5; *P*. *aeruginosa*-estA, C/N ratio = 83.2; and a 5g/mL rhamnose standard solution.

TLC was performed with the aim of observing whether the ratio of mono and di-RML was altered when EstA was overexpressed. Unlike Wilhelm et al. [13] and Tielen et al. [23], the overexpression of *estA* gene did not appear to be affected by the mono and di-RML ratio (Fig 4).

**Fig 5:**
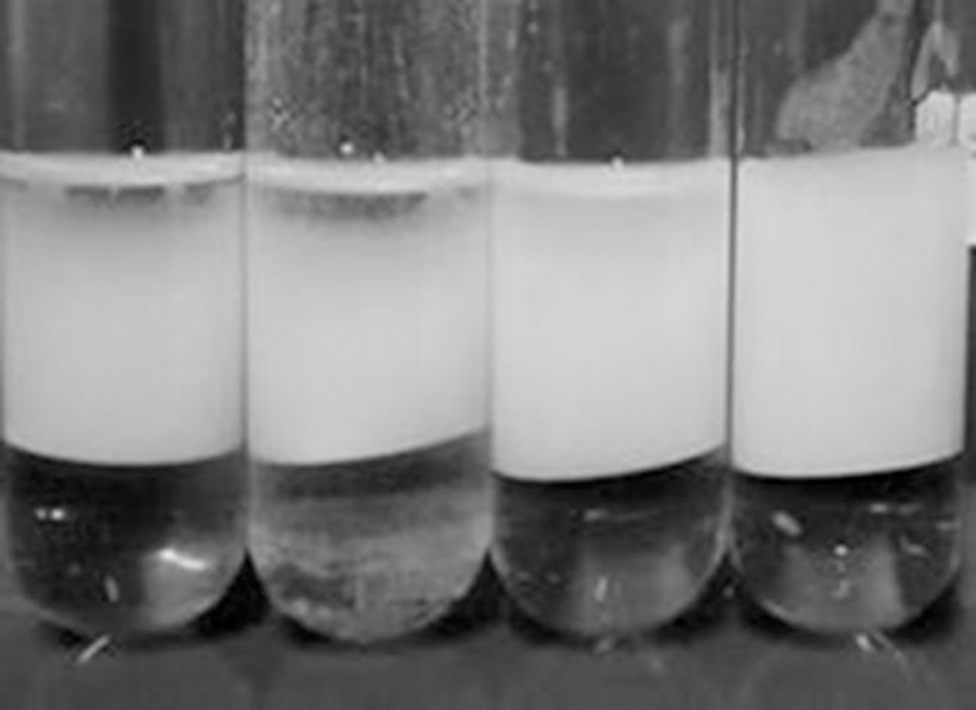
n-Hexadecane emulsion test. Last-day fermentation supernatant (free of glycerol) was diluted 1000 times and vigorously vortexed with equal parts of n-hexadecane, and left to stand for 24 hours. Then, the capacity of emulsifying the alkane was analyzed. From left to right: *P*. *aeruginosa* PAO1, with a C/N ratio of 17.5 and 83.2, then, *P*. *aeruginosa*-estA with a C/N ratio of 17.5 and 83.2 (mol/mol), respectively.

All the supernatants were able to fully emulsify n-hexadecane and keep the emulsification stable for more than a month. When the supernatants were diluted 1000 times, a small volume of unemulsified oil was observed at the top of the flask for the wild type, but not for the modified strain (Fig 5), which confirms the higher concentration and higher emulsifying capability of the supernatant from the recombinant *P*. *aeruginosa-estA*.

For the 83.2 medium, the mutant *P*. *aeruginosa-estA* produced a mixture that was able to reduce the surface tension of water (75 mN/m) to 29.33 mN/m and presented a CMC of 140 mg/L, and a CMD of 56.02. These values are compatible with the findings for RMLs in the literature, as reviewed by Lourith et al. [31]. Mendes et al. [32] produced RMLs under similar conditions, but using *P*. *aeruginosa* PA1, and found a CMC of 198 mg/L on the 7th fermentation day. However, glycerol consumption was not observed, and it is possible that there was some left over in the sample, which could have decreased the CMC value. The authors showed that after a purification step, the CMC of the RMLs produced was 25 mg/L, highlighting their promising use as a biosurfactant.

## Conclusions

In the present work, overexpression of *estA* gene, an outer-membrane esterase, was performed for the first time with the aim of achieving enhanced levels of RML production. It was shown that depending of the conditions, this strain was able to produce higher quantities of RML than the widely used wild-type strain *P*. *aeruginosa* PAO1. It was shown that both strains could produce significant quantities of RMLs using a low-cost medium, MSP-glycerol, which consists of just five simple, affordable components, including glycerol. No trace solution was used to increase productivity, as is often reported in the literature, and this medium was found to be one of the simplest and least costly media for RML production. A brief characterization of the molecules produced was performed, and presented surfactant properties compatible with those of interest to industry, such as the capacity to lower the surface tension of water and to emulsify oil (n-hexadecane). When compared with the bioprocesses described in the literature that use engineered strains, the strain constructed in this study showed great promise for further studies and its scaled up production for use in industry.
